# Dr. Davis, on an Hospital for Incurables

**Published:** 1807-04

**Authors:** J. B. Davis

**Affiliations:** Mint, Tower


### *To the Editors of the Medical and Physical Journal.*

GENTLEMEN,

AMIDST the immortal establishments of this great city, establishments which reflect brilliant recollections on the mind of every Englishman, and which will bear a glorious testimony to future ages of British philanthrophy; it is sincerely to be regretted, that no Institution has been created for the reception of a class of invalids termed Incurable. Buildings, in various parts of the metropolis, are consigned to the healing Art, efficacious resources are found within them for the relief and cure of diseases, and men of profound erudition and extensive practical knowledge, are chosen to instruct youth in all the branches of the sciences of medicine and surgery. In short, every method is adopted that superior talent can devise, and an unremitting attention to the executive part of medicine direct, to facilitate the acquirement of knowledge, and contribute to the relief of suffering humanity.

It is with the most heartfelt satisfaction that Philanthropists call to mind, the number of hospitals, infirmaries, and dispensaries that are supported in this city, a recollection justly endeared from a conviction of the

 happy happy effects they have produced on a large and useful part of Society. Those establishments have, with pride be it said, not only repaid, in an ample manner, the cares and solicitude of those who first gave birth to them, but of their successors also, who, emulating the noble example of their forefathers, have given a fuller developement to the principles on which they were founded, and even obtained by their liberal support, greater advantages from them than arose at former periods. Planted, if I may be allowed the expression, in a soil nurtured by the friends of humanity, they have many of them been patronized by the first and best of monarchs, and all have met with public protection.

Adverting then to the generous examples before us, is it rash to augur the success of an establishment for the relief of Incurables, and unreasonable to expect the union of sentiment and effort for whatever may tend to the general weal? I may, perhaps, be too sanguine, though I fondly hope to see another monument of public beneficence, which will be a new honour to the present age, and a shrine at which posterity will bow with respect, gratitude, and admiration.

Persons afflicted with complaints termed *Incurable*, cannot, from the peculiar nature of hospitals, and the vast number of patients admitted into them, receive that uninterrupted series of attention which their respective cases require, and which would be bestowed were there an asylum entirely devoted to the purpose. It is cruel, and very distressing to the feelings of a professional man, to dismiss a patient from the House of Succour, because he is, or rather seems to be, incurable. And, indeed, while a similar system is pursued, we oppose, if not preclude, the investigation of disease, which, although occult at one period, may become manifest at another: or, to say the least of it, it reflects a stigma on the dignity of the pofession, and seems as if we were luke-warm in promoting science.

A physician never loses a patient in these cases, but he laments more his igorance of the proper mode of treating him, than the incurableness of the complaint itself. Does it follow, I would beg leave to ask, that a disease is really incurable, because no person has hitherto been able to cure it? Certainly not. I would put no other construction on the term incurable, than that of not knowing a successful mode of treating a disease. How common an occurrence is it for patients to be discharged from hospitalstals, as *Incurables,* when the prescription of an old woman, an empiric, or Nature herself, works a cure. I can but think, when I reflect on this neglect of disease, and this extraordinary indifference, that we appear to renounce farther enquiries on the intricate points of pathology, and that we have reached the ne plus ultra of investigation.

Practitioners readily admit, by sad experience, the existence of defect somewhere; and most agree, that it is not so much in the art itself, as in the administration of it.

When we look at the catalogue of diseases that resist the means of cure our art at present possesses, then we see, in a still stronger point of view, the necessity of creating an Institution for the express purpose of endeavouring to afford relief to persons deemed *Incurable.* To enumerate all the complaints which would entitle individuals to the benefit of this charity would only hurt our recollections; I will select a few, of which the progress is not so rapid as to threaten a speedy dissolution, and consequently offering us the fairest opportunities of trying the effects of various medicines. The situation of phthisical patients, in this metropolis, is truly lamentable, and the number of individuals that fall victims to cancer, scrophula, anomalous and hectic fevers, epilepsy, dropsy, &c. is too striking not to call forth all our talent and humanity in seconding the plan I propose for their more minute investigation.

A spirit of research has signalized the philosophers of this country for a lapse of many years, delusive reasonings have yielded to experiment, and many modern improvements in medicine justify the presumption, that new discoveries will not fail shortly to be made. The progress in the arts of this isle has been very rapid, and has far surpassed that of rival nations; but the science of medicine has, in a more particular manner, been cultivated and brought to perfection. Emulation, formerly so common between the physicians of England and the Continent, and which always produced the most beneficial consequences, can now scarcely be said to exist. The spirit of investigation is chiefly confined to the eminent men of our own country, and will continue in it alone as long as arms are made the principal study of other nations.

The intention of this letter being only to suggest the outlines of a plan, a crude idea from which useful notions may be drawn, I leave to others to digest, and finish a

 system system that may redound to the credit of the profession. I could wish to see them ardent in the undertaking, forming a code of regulations for the treatment of persons that are not cured, and giving public lectures on the diseases that would fall within the limits of the institution. If once this desirable object could be accomplished the reflections of eminent men would be called forth, and experiment and theory would unite hand in hand to promote the advancement of science. And those persons, who thus devoted their talents to efface the opprobria of medicine, would soon acquire a merited celebrity; the annals of the times would record their glorious pursuits, and they would have the satisfaction of extending the limits of medical knowledge.

Persuaded, Gentlemen, of the interest that you and your Readers take in the grand objects of medicine, I entertain the hope that so respectable and learned a body will use its most strenuous efforts to found this establishment. Free from prejudice yourselves, I doubt not but you will strive to remove the prejudices of others, and encourage what may tend to benefit science.

I am thoroughly convinced, and I do not found my assertion on conjectural grounds, that many gentlemen, as well professional as others, have a fervent wish to see this desirable project realized. I have no idle desire, believe me, to witness the list of Philanthropic Institutions swell its pages; such motives only as I have described could inspire that feeling; and if the urgency of them could be impressed on the public mind, I doubt not but the one I recommend to your notice would be begun under the most favourable auspices.

What may be the result of delivering my opinion on this subject to the public, I cannot pretend to say, nor do I know how far it may meet your views. But be that as it may, the duty and the honour of the profession I exercise, prompt me to suggest an object of such importance, and I have laid on it the stress I thought it entitled to. I shall also have a satisfactory reflection at having devoted a few leisure moments, in favour of the unfortunate beings who are too often abandoned by their friends and physicians.

"Miseris succurrere disco."

I am, &c.

J. B. DAVIS, M. D.

